# Seborrheic keratosis in an adolescent: A rare presentation

**DOI:** 10.1002/ccr3.7697

**Published:** 2023-07-17

**Authors:** Pooria Zare, Mazaher Ramezani

**Affiliations:** ^1^ Students Research Committee Kermanshah University of Medical Sciences Kermanshah Iran; ^2^ Clinical Research Development Center, Imam Reza Hospital Kermanshah University of Medical Sciences Kermanshah Iran; ^3^ Molecular Pathology Research Center, Imam Reza Hospital Kermanshah University of Medical Sciences Kermanshah Iran

**Keywords:** adolescent, child, dermatology, keratosis, pathology, seborrheic

## Abstract

Seborrheic keratosis is a common benign epidermal lesion that typically occurs in the elderly population. Its occurrence in childhood and adolescence is rare and can pose a diagnostic challenge for clinicians and dermatopathologists. We report a case of a 16‐year‐old boy with multiple brown, black oval‐shaped stuck‐on lesions on his face, which were diagnosed as seborrheic keratosis based on histological findings. The patient had no significant medical history or family history of seborrheic keratosis. In this report, we discuss the differential diagnoses of seborrheic keratosis‐like lesions in childhood and adolescence and explain why they were not compatible with our case. We also review the available treatment options. Our case emphasizes the need for dermatologists to consider seborrheic keratosis in the differential diagnosis of cutaneous lesions in young patients.

## 1 INTRODUCTION

Seborrheic keratosis (SK) is a common benign epidermal lesion that is typically seen in middle‐aged and elderly individuals. It is characterized by well‐circumscribed, pigmented, waxy, and verrucous papules or plaques, commonly located on the face, trunk, and extremities excluding palms and soles. The incidence of SK increases with age, and it is rarely seen in children and adolescents.^1^, ^2^ Here we present a case of a 16‐year‐old boy with multiple brown, black oval‐shaped stuck‐on lesions on his face that was compatible with SK upon biopsy.

## 2 CASE PRESENTATION

**FIGURE 1:**
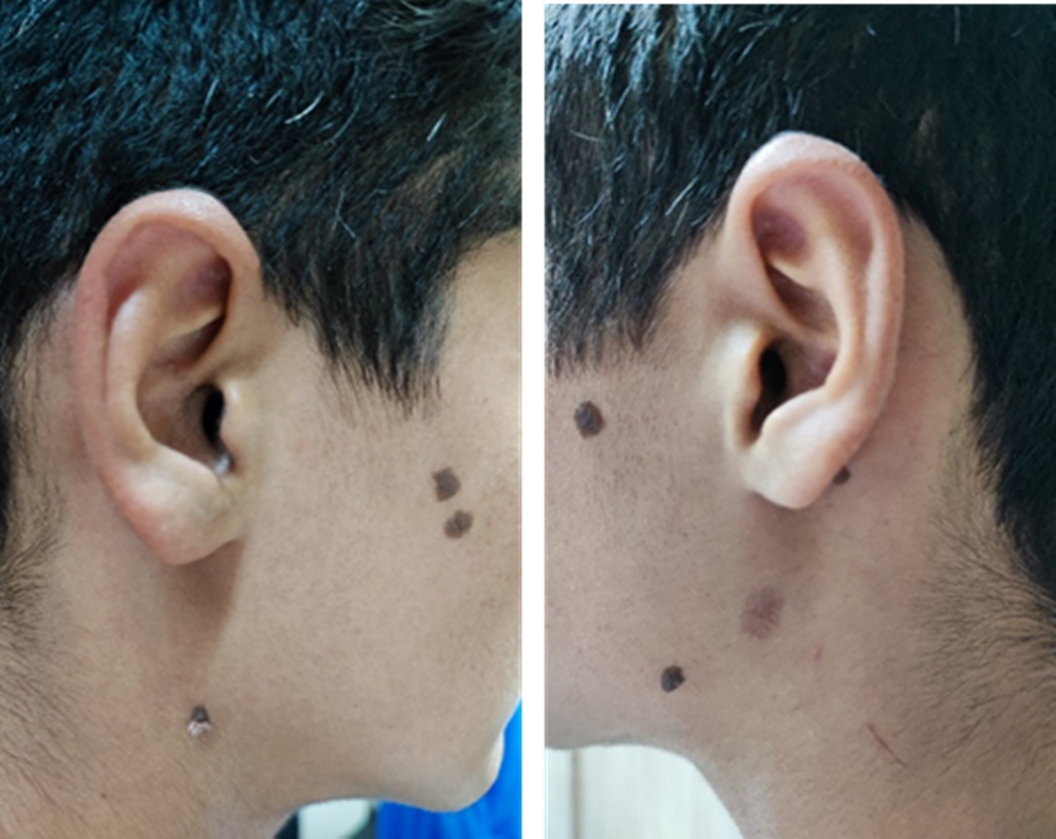
Multiple sharply demarcated, waxy, brown‐black, oval‐shaped papules and plaques with stuck‐on appearance.

**FIGURE 2:**
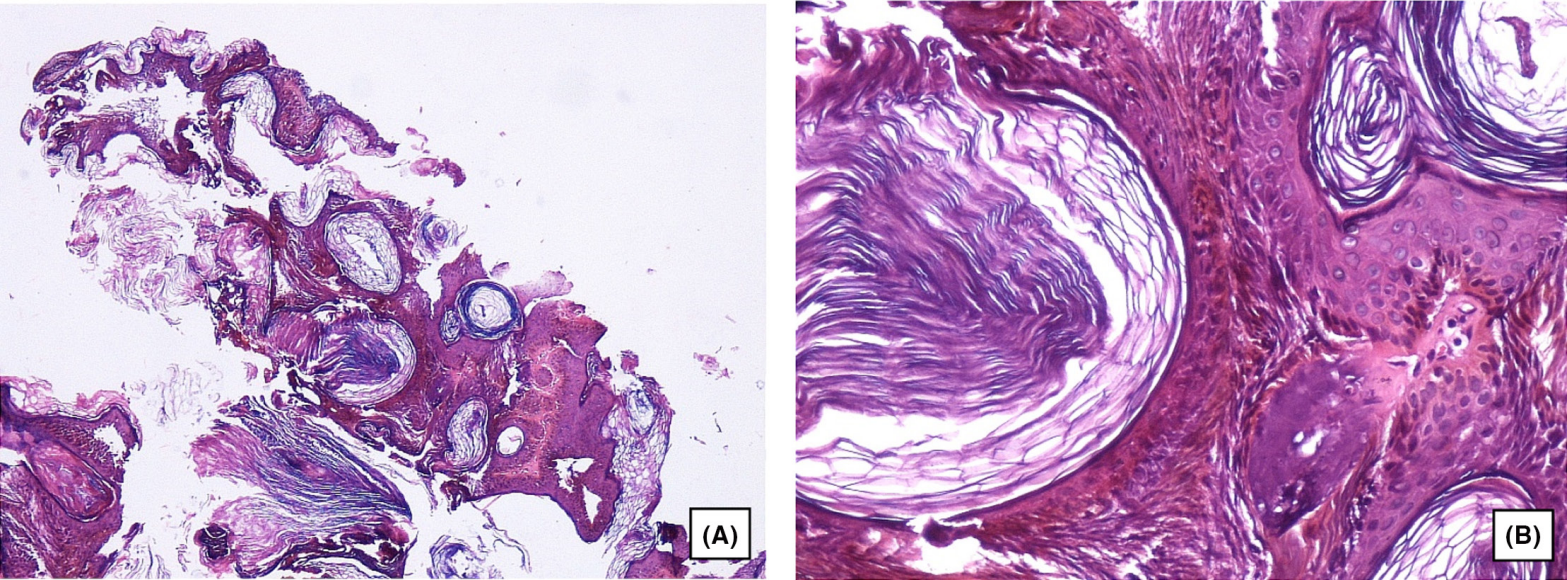
Histopathologic findings: (A) Low‐power view of hyperkeratosis with horn cysts and pseudocysts. (B) High‐power view of basaloid keratinocyte proliferation without dysplasia and horn pseudocyst formation. (Hematoxylin–Eosin stain).

A 16‐year‐old boy presented with multiple sharply demarcated, waxy, brown‐black, oval‐shaped lesions on his face measuring up to 1 cm in diameter (Figure 1), which had been present since he was about 7 years old. The lesions had not changed in size or appearance in the past 2–3 years. The patient had no other medical conditions, had not used any medication, and had no familial history of SK. A biopsy from one of the lesions was performed by a dermatologist and referred to our pathology department for examination, and upon microscopic findings, the lesions were compatible with SK. Basaloid keratinocyte proliferation without dysplasia and hyperkeratosis with horn pseudocyst formation were observed in the histological examination (Figure 2).

## 3 LITERATURE REVIEW

There have been few studies on SK in childhood or adolescence. Gill et al. studied 170 Australian people aged 15–30 years and mentioned that 23.7% of participants had at least one SK. Accordingly, they declared that the term *senile keratosis* is not a suitable synonym for *seborrheic keratosis*.^3^ In a case report by Ozbay et al., a 1‐year‐old boy presented with SK in his external auditory canal.^4^

## 4 DISCUSSION

Although SK is commonly seen in older adults, it can also occur in younger individuals, particularly in those with a familial history of the condition. Seborrheic keratosis is rarely seen in childhood or adolescence, and when present, it can pose a diagnostic challenge for clinicians and dermatopathologists. The differential diagnosis of SK in children and adolescents includes a variety of other benign and malignant skin lesions that may resemble SK clinically or histologically, such as acrokeratosis verruciformis of Hopf and epidermal nevus.^2^

In our case, the lesions were only located on the patient's face, specifically on his cheek, chin, and posterior to his left ear. This is in contrast to the acrokeratosis verruciformis of Hopf, which typically presents with lesions on the limbs and trunk. Furthermore, our case did not have the linear or whorled shape typically seen in epidermal nevi, making that diagnosis unlikely.

## 5 TREATMENT

Treatment for SK is typically not necessary, as the lesions are benign and do not pose any health risks. However, if the lesions are causing cosmetic concerns, they can be removed via cryotherapy, electrocautery, or surgical excision.^5^ In our case, the patient was referred to his dermatologist for proper treatment.

## 6 CONCLUSION

SK is a common cutaneous lesion found in elderly individuals; however, it should not be excluded from the differential diagnosis list for skin lesions in young people. Differential diagnoses for SK in the young age group include acrokeratosis verruciformis of Hopf and epidermal nevi, but our case was not compatible with these diagnoses due to the location and shape of the lesions. Treatment for SK is typically not necessary but can be done for cosmetic reasons.

## AUTHOR CONTRIBUTIONS

**Pooria Zare:** Data curation; investigation; writing – original draft. **Mazaher Ramezani:** Conceptualization; supervision; writing – review and editing.

## FUNDING INFORMATION

None.

## CONFLICT OF INTEREST STATEMENT

None.

## ETHICS STATEMENT

This case report is ethical according to the world medical association declaration of Helsinki.

## CONSENT STATEMENT

Written informed consent is obtained from the patient to publish this report.

## Data Availability

The data that support the findings of this study are available on request from the corresponding author. The data are not publicly available due to privacy or ethical restrictions.
